# Recent Advances in Aging and Immunosenescence: Mechanisms and Therapeutic Strategies

**DOI:** 10.3390/cells14070499

**Published:** 2025-03-27

**Authors:** Shuaiqi Wang, Tong Huo, Mingyang Lu, Yueqi Zhao, Jianmin Zhang, Wei He, Hui Chen

**Affiliations:** 1 Department of Immunology, CAMS Key Laboratory T-Cell and Cancer Immunotherapy, Institute of Basic Medical Sciences, Chinese Academy of Medical Sciences and School of Basic Medicine, Peking Union Medical College, State Key Laboratory of Common Mechanism Research for Major Diseases, Beijing 100005, China; b2023005058@pumc.edu.cn (S.W.); huotong1998@pumc.edu.cn (T.H.); b2022005055@student.pumc.edu.cn (M.L.); s2022005053@student.pumc.edu.cn (Y.Z.); zhangjianmin@ibms.pumc.edu.cn (J.Z.); 2 Changzhou Xitaihu Institute for Frontier Technology of Cell Therapy, Changzhou 213000, China

**Keywords:** aging, cellular senescence, immunosenescence, SASP, senolytic

## Abstract

Cellular senescence is an irreversible state of cell cycle arrest. Senescent cells (SCs) accumulate in the body with age and secrete harmful substances known as the senescence-associated secretory phenotype (SASP), causing chronic inflammation; at the same time, chronic inflammation leads to a decrease in immune system function, known as immunosenescence, which further accelerates the aging process. Cellular senescence and immunosenescence are closely related to a variety of chronic diseases, including cardiovascular diseases, metabolic disorders, autoimmune diseases, and neurodegenerative diseases. Studying the mechanisms of cellular senescence and immunosenescence and developing targeted interventions are crucial for improving the immune function and quality of life of elderly people. Here, we review a series of recent studies focusing on the molecular mechanisms of cellular senescence and immunosenescence, the regulation of aging by the immune system, and the latest advances in basic and clinical research on senolytics. We summarize the cellular and animal models related to aging research, as well as the mechanisms, strategies, and future directions of aging interventions from an immunological perspective, with the hope of laying the foundation for developing novel and practical anti-aging therapies.

## 1. Introduction

Population aging is currently one of the major global challenges [1]. With the intensification of population aging, delaying aging and improving the quality of life for elderly people have become important tasks. Aging is a multifactorial process driven by various intrinsic and extrinsic factors, including genomic instability, telomere attrition, epigenetic alterations, loss of proteostasis, disabled macroautophagy, deregulated nutrient sensing, mitochondrial dysfunction, cellular senescence, stem cell exhaustion, altered intercellular communication, chronic inflammation, and dysbiosis [2]. These factors are closely related to organismal aging, and research has shown that inducing them can accelerate aging, while intervening in them can slow down, halt, or even reverse the aging process [2]. Thoroughly studying these aging factors to elucidate the mechanisms of aging can help identify interventions to delay aging, such as caloric restriction, nutritional interventions, and gut microbiota transplantation, as well as clinical treatments for aging-related diseases, including senolytics, stem cell therapy, and antioxidant and anti-inflammatory treatments. These approaches can mitigate aging and aging-related diseases, thereby achieving healthy aging and longevity [3,4,5].

Among these factors, cellular senescence is a key contributor to organismal aging. Targeting senescent cells (SCs) holds promise for developing novel and practical anti-aging therapies [6]. Cellular senescence is an irreversible state of cell cycle arrest caused by various factors, such as DNA damage and telomere shortening [7,8]. Additionally, the process whereby immune system function gradually declines or becomes dysregulated with human aging is known as immunosenescence [9]. Although considerable variability in aging exists among individuals, the aging process generally involves chronic inflammation, tissue homeostasis disorders, and dysfunction of the immune system and organ functions, [2] readily causing cardiovascular, metabolic, autoimmune, and neurodegenerative diseases associated with aging [5,10,11,12,13]. Existing research indicates that transplanting SCs into young mice induces bodily dysfunction, while transplanting them into aged mice exacerbates aging and increases the risk of death [6]. This suggests that SCs accelerate organismal aging. The specific reason is that SCs release the senescence-associated secretory phenotype (SASP) into the tissue, promoting chronic inflammation and inducing senescence in surrounding tissue cells and immune cells [14]. SCs and chronic inflammation interact and crosstalk, forming a vicious cycle of inflammation and aging. Therefore, in-depth research into the key characteristics and underlying mechanisms of cellular senescence, immunosenescence, and inflammation, identifying drug intervention targets, and developing targeted interventions can help mitigate aging and aging-related diseases, thereby promoting healthy aging in the elderly.

**Figure 1:**
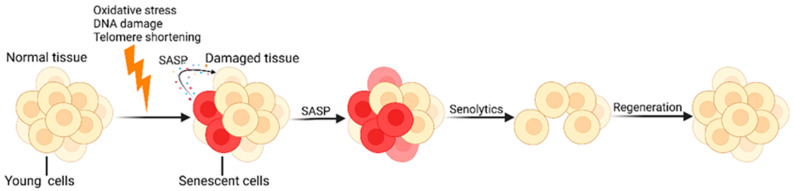
Cellular senescence and senolytics. SCs continuously produce numerous pro-inflammatory molecules and tissue-remodeling molecules, known as the SASP, which further accelerates the aging process. Senolytics promote the regeneration of new healthy cells by identifying and clearing SCs. Created with BioRender.com (accessed on 10 May 2024).

In recent years, based on the establishment of a series of aging-related cellular and animal models (Table 1), the latest research has revealed the molecular mechanisms of cellular senescence and immunosenescence and the body’s regulation of aging from an immune response perspective. Moreover, based on new mechanisms, strategies targeting the elimination of SCs have become a promising treatment method for alleviating aging and age-related diseases. Initially, scientists utilized transgenic strategies to clear accumulated SCs in mice, which could treat age-related diseases and extend healthspan [15,16]. Later, it was discovered that some small-molecule senolytic drugs that target proteins in senescent cell antiapoptotic pathways (SCAPs) can selectively kill SCs (Figure 1) [17,18]. Currently, effective, safe, and selective immunotherapy approaches targeting SCs are gradually becoming a promising treatment method. Some research teams have already developed senolytic CAR T cells [19], senolytic vaccines [20], and immune checkpoint blockade (ICB) therapies to achieve the clearance of SCs [21]. In this review, we revisit a series of significant research findings and advances in this field over recent years.

## 2. Molecular Mechanisms of Cellular Senescence and Immunosenescence

In normal physiological processes, cellular senescence acts as a crucial tumor-suppressive mechanism by preventing the proliferation of precancerous cells, thus inhibiting the onset of tumors and playing a role in tissue damage repair [35,36]. However, under pathological conditions, SCs continuously produce numerous proinflammatory and tissue remodeling molecules, causing chronic inflammation and further accelerating the aging process and the onset of various age-related diseases (Figure 1) [14,36,37]. Alongside human aging, the function of the immune system gradually declines; during immunosenescence, reductions in the number and function of immune cells, weakened immune memory and responses, and a replacement of naive T and B cells by memory cells are observed [14,38]. In a healthy body, SCs are detected and cleared by immune cells [25,30,39]; thus, specifically activating the immune system can improve aging and age-related diseases [28]. However, senescent immune cells not only fail to clear SCs and inflammatory factors but also accelerate the aging of the body, thereby forming a vicious cycle of inflammation and aging [33]. Previous research has shown that various factors, such as genetics, exercise, nutrition, the gut microbiota, and pathogen infections, significantly affect the process of immunosenescence [4,40,41,42], but the specific underlying mechanisms need further exploration.

In recent years, a series of aging-related cellular and animal models have been established, and significant progress has been achieved in understanding the molecular mechanisms of cellular senescence, immunosenescence, and the regulation of aging by the immune system.

### 2.1. Molecular Mechanisms of Cellular Senescence

#### 2.1.1. The cGAS-STING Pathway Promotes Age-Related Inflammation and Accelerates Aging

The cGAS-STING signaling pathway can recognize abnormal DNA within cells, as well as DNA fragments from invading pathogens, producing type-I interferons and other inflammatory cytokines to exert immune functions [43]. In recent years, activation of the cGAS-STING pathway has been reported to be involved in regulating the cellular aging process [44,45]. However, whether the cGAS-STING pathway can directly cause cellular senescence in tissues and age-related inflammation in the body remains unclear.

In August 2023, Andrea Ablasser et al. from the Swiss Federal Institute of Technology Lausanne published a study in *Nature* [29]. Using a natural aging mouse model, they discovered that administering the STING inhibitor H-151 or knocking out *STING* could significantly improve age-related inflammation. Furthermore, they investigated the impact of the cGAS-STING pathway on brain aging. They found abnormalities in mitochondrial morphology in the microglia of aged mice, with mitochondrial DNA released into the cytoplasm to activate the cGAS-STING pathway.

This study, which started with naturally aging mice, revealed that activation of the cGAS-STING pathway is a major promoter of age-related inflammation. Targeting the cGAS-STING pathway may become a viable strategy for improving or even reversing age-related damage.

#### 2.1.2. Endogenous Retroviruses Resurrected in the Human Genome Drive Aging

Extensive research has been conducted on the regulation of aging by protein-coding genes, but the regulatory role of noncoding regions of the genome in aging has also gradually attracted increasing attention. Reports have revealed that silent long-interspersed element-1 (*LINE1*) retrotransposons are activated during the aging process and trigger an innate immune response associated with aging phenotypes [46,47].

Endogenous retroviruses (*ERVs)* are another type of transposable element that is dormant within our genome [48]. In January 2023, Guang-Hui Liu et al. from the Institute of Zoology at the Chinese Academy of Sciences published a research paper in the journal *Cell* revealing that *ERVs* are reactivated during the aging process and promote aging [23]. The researchers used various aging systems to discover that epigenetic derepression in SCs leads to the transcriptional activation and translation of viral proteins from a young *ERV* subfamily, *HERVK* (*HML-2*), resulting in the formation of retrovirus-like particles (RVLPs). On the one hand, the reverse transcription products of *HERVK* activate the cGAS-STING pathway, inducing cellular senescence and inflammation. On the other hand, the RVLPs released by aging cells are transmitted between cells and amplify aging signals, ultimately inducing senescence in young cells.

This study first described the reactivation of a young *ERV* subfamily during the cellular aging process, proposed the theory that ancient virus revival mediates the programmed and infectious aspects of aging, and innovatively developed multidimensional intervention strategies to delay aging by blocking the revival and spread of *ERVs*.

#### 2.1.3. The CCF-TXNRD1-cGAS Axis Regulates Age-Related Inflammation

As aging progresses, the integrity of the nuclear envelope is compromised, allowing a large amount of cytoplasmic chromatin fragments (CCFs) to leak from the nucleus into the cytoplasm. These CCFs promote the SASP in aging cells by activating the cGAS-STING pathway [49,50].

Thioredoxin reductases (TXNRDs) can inhibit oxidative stress and are among the most important antioxidant proteins in cells [51]. The cytoplasmic isoform of TXNRDs, TXNRD1, is considered to play a role in tissue aging related to its enzymatic activity, as oxidative damage can lead to tissue aging and inflammation [52]. However, research published in *Nature Aging* by Rugang Zhang’s team at the MD Anderson Cancer Center revealed that TXNRD1 colocalizes with cGAS in SCs, promoting the binding of cGAS to DNA, which in turn drives the SASP and inflammation [53].

Researchers found that knocking down *TXNRD1* in SCs or using its specific inhibitor Tri-1 significantly reduced SASP levels. Further studies using two different TXNRD1 inhibitors showed that only inhibiting the interaction between TXNRD1 and cGAS could reduce SASP levels, thereby indicating that the regulation of the SASP by TXNRD1 does not depend on its enzymatic activity but rather on its interaction with cGAS. In the future, strategies targeting the interaction between TXNRD1 and cGAS hold significant potential for the development of clinical treatments for senescence and age-related diseases.

#### 2.1.4. The TORC1-S6K-Syx13 Signaling Pathway Regulates Aging Through the Endolysosomal System

Rapamycin modulates the aging process by acting on downstream autophagy and S6 protein kinase (S6K) through target of rapamycin complex 1 (TORC1) [54]. Although early studies have shown that the absence of S6K can extend the lifespan of mice [55], the specific mechanisms of action involved remain unclear.

In February 2024, Linda Partridge et al. published research in the journal *Nature Aging* that revealed the mechanisms by which S6K regulates aging [56]. Using fruit flies as a model, the research team identified the adipose tissue of adult fruit flies as the key tissue where the TORC1-S6K pathway regulates aging. By inhibiting S6K activity in adipose tissue, they were able to reduce age-related inflammation mediated by the IMD pathway in the adipose tissue of elderly fruit flies and improve immunosenescence caused by aging. Furthermore, they discovered that syntaxin 13 (Syx13), which is associated with cell membrane fusion, mediates the effects of the TORC1-S6K signaling pathway on the morphology of endolysosomes and inflammation, thereby showing that the TORC1-S6K-Syx13 signaling pathway regulates aging and age-related inflammation through the endolysosomal system.

This study established the endolysosomal system as a novel cellular mechanism that mediates the regulatory effects of rapamycin and S6K on immunosenescence and lifespan, thus providing a new direction for future research and treatment related to aging.

### 2.2. Molecular Mechanisms of Immunosenescence

#### 2.2.1. Increased Expression of CISH in Activated T Cells in Elderly Individuals Causes Immunosenescence

The two main hallmarks of T cell senescence are immunodeficiency and inflammaging [57]. Inflammaging is a systemic chronic inflammatory state that occurs without obvious infection and is capable of causing widespread tissue dysfunction, frailty, and premature death [14,58,59]. However, the mechanisms underlying inflammaging are currently not well understood.

Previous studies have indicated that the majority of the effector molecules involved in T-cell-mediated inflammation are cytokines. Recently, a study published in *Nature Aging* by Jorg J. Goronzy et al. revealed that activated T cells in elderly individuals can mediate strong inflammatory responses by releasing large amounts of mitochondrial DNA [60]. They found that increased expression of cytokine-inducible SH2-containing protein (CISH) promotes the proteasomal degradation of ATP6V1A, an essential component of the proton pump V-ATPase, leading to a diminished lysosomal acidification capacity and thus inhibiting lysosomal degradation functions. A decrease in lysosomal function prevents the clearance of upstream autophagosomes and late endosomes, causing them to accumulate and fuse within the cell to form amphisomes, which subsequently merge with the cell membrane and release their contents, including mitochondrial DNA, to the exterior of the cell. By further utilizing murine infection and immunization models, the researchers have shown that silencing CISH in mouse T cells reduces the serum mitochondrial DNA and inflammatory cytokine levels while enhancing virus clearance and antibody production.

This study suggested that downregulating CISH expression in T cells is a promising strategy for enhancing lysosomal function, alleviating inflammaging in elderly individuals, and ultimately boosting immunity.

#### 2.2.2. IL-33 Induces Thymic Involution-Associated Naive T Cell Aging

The thymus plays a crucial role in the development and function of the immune system, and thymic involution is one of the main hallmarks of T cell senescence [57,61]. Severe infectious diseases often cause acute thymic involution, [42,62] which triggers immunosuppression [63]. However, the exact molecular basis of acute thymic involution during severe infections and the related functional impairments in T cell senescence are not yet fully understood.

In November 2022, Xiaojun Chen et al. published an article in *Nature Communications* revealing that IL-33 induces immunosuppression by causing thymic involution associated with naive T cell functional impairment and aberrant expression of aging-related genes [64]. Using mouse disease models of schistosomiasis and sepsis, the research team discovered that IL-33 is a critical factor that causes host thymic involution and T cell senescence. Further studies revealed that IL-33 induces the excessive production of medullary thymic epithelial cell (mTEC) IV (thymic tuft cells) in a *Pou2f3*-dependent manner, disrupting the mTEC/cortical TEC (cTEC) compartment and thereby leading to thymic involution. Knockout of IL-33 or its receptor ST2, as well as treatment with an IL-33 neutralizing antibody, could eliminate host thymic involution, thereby restoring T cell responses and ultimately enhancing host infection resistance.

In summary, reversing thymic involution is a potential therapeutic strategy that can restore T cell immune responses in order to better control severe infections.

#### 2.2.3. Commensal Bacteria Induce the Aging of Germinal Center B Cells in the Gut

Many studies have confirmed that aging in organisms is accompanied by changes in the gut microbiota composition, leading to age-related functional decline and the development of diseases [65,66,67]. However, the mechanisms and reasons for these age-related changes in the gut microbiota composition are still not well understood.

In May 2023, Eiji Hara et al. revealed that the presence of commensal bacteria can induce aging in B cells located in the germinal center (GC) of the gut [68]. The researchers initially used bioluminescence imaging (BLI) and single-cell RNA sequencing (scRNA-seq) to analyze aging markers in specific pathogen-free (SPF) and germ-free (GF) mice. They found that only the GC B cells in the ileal lymphoid follicles of aging SPF mice exhibited significant signs of aging, indicating that the presence of gut bacteria could induce B cell aging by upregulating the expression of *p16INK4a* and *p19ARF*. Further studies revealed that lipopolysaccharide (LPS) from gram-negative bacteria could penetrate into ileal tissues. LPS-treated cultured B cells induced B cell hyperproliferation and upregulated *p16INK4a* expression, along with causing DNA damage in B cells.

Overall, this study revealed that with increasing age, the likelihood of gram-negative bacteria invading the ileum increases, which in turn causes B cell aging. The aging of GC B cells reduces the production and abundance of IgA, thereby affecting the composition of the gut microbiota.

### 2.3. Regulation of Aging by the Immune System

#### 2.3.1. CD4^+^ CTLs Eliminate SCs by Targeting Cytomegalovirus Antigens

Previous studies have focused on clearing SCs using pharmacological or genetic approaches, which have significant side effects [17,69]. Considering that SCs produce SASP components, they could serve as targets for the immune system. However, researchers have not yet clearly determined how the immune system combats SCs in the human body.

Recently, a research team led by Shadmehr Demehri at Massachusetts General Hospital and Harvard Medical School published an article in *Cell* titled “Cytotoxic CD4^+^ T cells eliminate senescent cells by targeting cytomegalovirus antigen” [25]. The researchers studied human skin samples of various ages and found that the chemokine CXCL9 expressed by keratinocytes recruits CD4^+^ CTLs to the skin. Senescent fibroblasts express HLA-II and human cytomegalovirus glycoprotein B (HCMV-gB). HLA-II then presents HCMV-gB as an antigen on the fibroblast surface, which can be directly targeted and cleared by CD4^+^ CTLs.

This study revealed the interaction between the human immune system and viruses, providing a theoretical basis for designing immunotherapies targeting HCMV antigens to clear SCs.

#### 2.3.2. Immunosenescence Drives Aging in Solid Organs

SCs are key drivers of organismal aging, and clearing these cells can delay or alleviate many age-related diseases [6]. Therefore, identifying which types of SCs contribute most significantly to aging and targeting them as a priority for treatment remain critical issues.

Laura J. Niedernhofer et al. published a study in *Nature* revealing that senescent immune cells are the most perilous type of SCs, accelerating the aging of other organs and thus promoting systemic aging [33]. To determine the impact of immunosenescence on organismal aging, the researchers selectively deleted the *Ercc1* gene, which encodes a key DNA repair protein, in the hematopoietic cells of mice [70]. This deletion increased the burden of endogenous DNA damage exclusively within the immune system, leading to premature aging of the immune system alone. However, these mice also exhibited increased aging and damage in non-lymphoid organs. Furthermore, transplanting spleen cells from *Ercc1* knockout mice or aged wild-type mice into young mice accelerated aging in the recipients, while transplanting young immune cells mitigated aging. This result further indicates that senescent immune cells can promote systemic aging.

In summary, this study suggested that the senescence of immune cells may be the most detrimental to the organism. Therefore, senescent immune cells have become a key therapeutic target for extending a healthy lifespan.

#### 2.3.3. IgG Leads to Adipose Tissue Fibrosis

As early as 2020, a research team led by Professor Li Qiang at Columbia University first confirmed that adipose tissue is the tissue initiating aging through metabolomic analysis of multiple tissues in mice [71]. Adipose tissue plays a central role in longevity, and interventions targeting adipose tissue may influence the lifespan [72].

Unlike IgA or IgM, IgG has an especially long half-life due to its unique recycling mechanism [73]. Beyond traditional immune functions, the role of IgG in aging and metabolism remains unclear. In February 2024, Professor Li Qiang’s research team published an article in *Cell Metabolism* revealing that IgG is an aging factor that causes fibrosis in adipose tissue and metabolic decline [74]. The researchers discovered a significant enrichment of IgG in the visceral fat of aging mice through quantitative proteomics and found that administering exogenous IgG to mice on caloric restriction (CR) could reverse the improvements in adipose tissue function induced by CR. Further mechanistic studies showed that IgG activates macrophages via the Ras signaling pathway and induces fibrosis in white adipose tissue (WAT) through the TGF-β/SMAD pathway, thereby impairing the metabolic function of adipose tissue. The neonatal Fc receptor (FcRn) is the recycling receptor for IgG in macrophages [73], and conditional knockout of this receptor can prevent the accumulation of IgG during aging, thereby extending a healthy lifespan.

In summary, this study revealed that IgG begins to accumulate systemically early in aging, particularly in adipose tissue, leading to fibrosis and metabolic damage in the tissue. Therefore, intervening in the accumulation of IgG represents a viable therapeutic strategy for delaying aging.

#### 2.3.4. APCs Transfer Telomeres to T Cells to Protect T Cells from Aging

As the number of cell divisions increases, telomeres become progressively shorter, eventually leading to cellular senescence [75]. Although T cells can utilize telomerase to mitigate telomere shortening caused by rapid clonal expansion, the activation of telomerase is not sufficient to prevent T cell exhaustion, ultimately still resulting in the production of senescent T cells [76,77,78].

Recently, researchers at University College London (UCL) discovered that some T cells can extend their own telomeres by acquiring telomeres from extracellular vesicles (EVs) secreted by antigen-presenting cells (APCs) [78]. Further mechanistic studies revealed that when some T cells interact with APCs, the APCs degrade shelterin to provide telomeres. These telomeres are then cleaved by the telomere trimming factor TZAP and transferred in EVs at the immunological synapse. The telomere vesicles retain the Rad51 recombinase, which enables the telomeres to fuse with the ends of the T cell chromosomes, extending them by an average of approximately 3000 base pairs.

Overall, this study revealed that the transfer of telomeres from APCs to T cells can protect T cells from senescence. When T cells acquire telomeres from APCs during antigen presentation, they transition to a state similar to central long-lived memory T cells, providing long-term immune protection for the organism.

## 3. Strategies for Intervening in Aging

### 3.1. Intervening in Aging with Small-Molecule Senolytic Drugs

Aging is a defining characteristic of various age-related human diseases, and targeting the elimination of SCs has recently become a promising therapeutic approach to alleviate aging and age-related diseases. The use of transgenic strategies to clear SCs has been proven to delay aging, treat age-related diseases, and extend healthy lifespan [15,16,35]. Baker et al. utilized the aging biomarker *p16Ink4a* to design a novel transgenic mouse model, *INK-ATTAC*, that induces the elimination of *p16Ink4a*-positive SCs upon drug administration, and showed that using genetic methods to eliminate SCs can significantly delay the onset of age-related diseases and prolong healthy lifespan [15]. Subsequently, scientists have attempted to selectively kill SCs using small-molecule senolytic drugs while sparing normal cells [18]. SCs are protected from apoptosis by SCAPs [17], and targeting proteins within SCAPs with small-molecule senolytic drugs can selectively kill SCs; these effector proteins are referred to as senolytic targets [79]. To date, several classes of senolytics have been identified, including natural and synthetic molecules. Natural molecules include dasatinib, quercetin, fisetin, and piperlongumine, while synthetic molecules include Navitoclax, EF24, UBX0101, A1331852, and A1155463 [17,79].

#### 3.1.1. Quercetin

Quercetin has a wide range of biological activities, such as antioxidant, anticancer, and anti-inflammatory effects [80]. In 2015, quercetin was first discovered to be a senolytic that can effectively kill senescent human endothelial cells and mouse bone marrow-derived mesenchymal stem cells (BM-MSCs) [18]. In 2019, researchers at the Mayo Clinic observed that the combination of dasatinib and quercetin (D&Q) successfully cleared SCs in patients with diabetic nephropathy, significantly reducing the burden of SCs in adipose and skin tissues [81]. Furthermore, a recent article published in *Science* reported that the neural innervation of the heart weakens with age, but this effect can be reversed with treatment using the D&Q senolytics combination [82]. However, quercetin must be used in conjunction with dasatinib to exhibit effective senolytic activity [18,81].

#### 3.1.2. Fisetin

Fisetin is another flavonoid that has shown strong antitumor activity by inhibiting cancer cell proliferation and inducing apoptosis in cancer cells [83]. Research has shown that the anti-proliferative and pro-apoptotic effects of fisetin are limited to cancer cells and have a much weaker impact on normal cells [84]. In 2017, James L. Kirkland et al. first discovered that fisetin selectively induces apoptosis in SCs. It induced apoptosis in senescent but nonproliferating human umbilical vein endothelial cells (HUVECs) without affecting proliferating HUVECs, making it a cell-specific senolytic [85]. Later, Matthew J. Yousefzadeh et al. conducted in vivo studies on the effects of fisetin on aging in aged mice, confirming the senolytic activity of fisetin, which can reduce senescence markers in multiple tissues [86].

Additionally, ongoing basic research and clinical studies are further exploring senolytics such as quercetin, dasatinib, fisetin, piperlongumine, and EF24. Several clinical trials involving senolytics are currently underway or planned (Table 2), and these senolytics have provided evidence of clearing SCs in various pathologies.

### 3.2. Intervention in Aging Through Immunological Means

Although traditional small-molecule senolytics have shown promising results in eliminating SCs and alleviating age-related diseases, these small-molecule senolytics exhibit imperfect specificity and potential toxicity to healthy tissues [87,88], limiting strategies for selectively eliminating SCs due to their toxicity and lack of efficacy. Therefore, the need for selective, effective, and safe therapeutic approaches targeting SCs has driven the development of new treatment paradigms for aging and age-related diseases. In recent years, innovative strategies using immunological interventions such as chimeric antigen receptor (CAR) T cells, senolytic vaccines, and immune checkpoint blockade (ICB) have been proposed and have achieved significant therapeutic effects on animal models.

#### 3.2.1. Senolytic CAR T Cells Reverse Age-Related Pathologies

Reengineering patients’ own T cells to selectively target and eliminate tumor cells has cured patients with otherwise untreatable hematological cancers [89]. However, evidence from both basic and clinical research has highlighted the potential of CAR T therapy to go beyond oncology, addressing autoimmune diseases, chronic infections, cardiac fibrosis, age-related diseases, and other conditions [90,91].

In 2020, a team led by Scott W. Lowe at the Memorial Sloan Kettering Cancer Center published an article in *Nature* proposing a method to treat age-related pathologies using CAR T cells [19]. Researchers identified urokinase-type plasminogen activator receptor (uPAR) as a specific SCs surface marker and developed uPAR CAR T cells. They found that these cells could safely and effectively clear SCs in several induced young mouse models and reverse liver fibrosis in a liver disease model. This study confirmed the potential of senolytic CAR T cells to treat age-related diseases and provided a new direction for the future treatment of age-related conditions.

Recently, Scott W. Lowe’s team published their latest research findings in *Nature Aging* [92]. They demonstrated that uPAR-positive SCs accumulate during the aging process in mice and can be safely targeted and eliminated by CAR T cells. Treatment with CAR T cells targeting uPAR improved the mobility of aged mice and ameliorated metabolic dysfunction in both aged mice and mice on a high-fat diet (HFD) without causing any tissue damage or toxic effects. More importantly, due to the memory capacity and longevity of T cells, this senolytic CAR T cells therapy requires only a single administration to achieve long-term therapeutic and preventive effects.

#### 3.2.2. NKG2D-CAR T Cells Eliminate SCs in Aged Animals

NKG2D ligands (NKG2DLs), which include MICA, MICB, and ULBP1-6, are highly expressed in tumor cells, and numerous cancer therapies targeting these ligands have entered clinical trials without the discovery of serious side effects [93,94,95]. In humans, NKG2DLs also act as triggers for the NK cell-mediated killing of SCs [96]. Therefore, in anti-aging research prioritizing safety, NKG2DLs are undoubtedly ideal targets for the elimination of SCs.

In August 2023, Xudong Zhao’s team published a cover article in *Science Translational Medicine* detailing NKG2D-CAR T cells targeting NKG2DLs as effective and safe senolytic agents [28]. Researchers first reported the significant upregulation of NKG2DLs in various stress-induced SCs and then engineered NKG2D-CAR T cells that recognize NKG2DLs. In vitro experiments showed that NKG2D-CAR T cells significantly killed SCs induced by DNA damage, replicative exhaustion, oncogene activation, or tumor suppressor gene inactivation in a dose-dependent manner. In vivo, the researchers developed mNKG2D-CAR T cells for mice and hNKG2D-CAR T cells for macaques, which significantly reduced the number of SCs in aging animals and did not cause severe side effects.

In summary, this study, based on the emerging CAR T immunotherapy for clearing SCs, demonstrated the feasibility of using NKG2DLs as anti-aging targets. It lays the foundation for developing more effective, safe, and precise anti-aging treatment methods.

#### 3.2.3. Senolytic Vaccination to Remove SCs from the Body

Traditionally, vaccines have primarily been used for the prevention of infectious diseases and the treatment of cancer [97,98]. In 2020, a research paper published in *Nature Communications* reported for the first time the use of a vaccine as a therapeutic tool to eliminate SCs [32].

Senescent T cells, which increase with age, are defined as CD4^+^ CD44^high^ CD62L^low^ PD-1^+^ CD153^+^ cells and accumulate in the visceral adipose tissue (VAT) of obese individuals, directly correlating with the development of various diseases [99]. Researchers developed a CD153-CpG vaccine and confirmed that vaccination increased and sustained anti-CD153 antibody levels for several months. They found that the number of aging T cells in the VAT of HFD-induced obese mice vaccinated with the CD153-CpG vaccine was significantly reduced, and these mice exhibited improved glucose tolerance and lower insulin resistance. Complement-dependent cytotoxicity (CDC) assays further revealed that mouse IgG2 antibodies produced in mice vaccinated with the CD153-CpG vaccine successfully reduced the number of senescent T cells [32]. This study innovatively developed a senolytic vaccination capable of removing senescent T cells from the body, demonstrating the new potential applications of vaccines.

Subsequently, Tohru Minamino et al. identified a target for senolytic therapy—glycoprotein nonmetastatic melanoma protein B (GPNMB) [20]. GPNMB is a transmembrane protein that is enriched on the surface of some SCs. The research team developed a peptide vaccine for GPNMB. Upon injection, the vaccine-generated antibodies bind only to the GPNMB protein on the surface of SCs, thereby marking these cells for destruction. Then, these antibodies induce antibody-dependent cell-mediated cytotoxicity (ADCC) to kill SCs, an effect that was validated in models of atherosclerosis and aging mice.

In summary, these studies suggest that vaccines may be a viable tool for the treatment of aging and age-related diseases. Senolytic vaccination could become a new and suitable therapeutic approach, although its clinical application requires further assessment and the management of safety.

#### 3.2.4. Blocking PD-L1/PD-1 Improves Aging Phenotypes

Many studies have shown that the immune system can clear SCs induced by various causes [25,30,39]. However, despite this capability, SCs still accumulate in various tissues and organs with aging [100,101]. Currently, little is known about the molecular mechanism underlying the accumulation of SCs.

In the study of cancer, scientists have discovered that the immune surveillance of tumor cells is negatively regulated by immune checkpoints [102,103]. In November 2022, a team led by Makoto Nakanishi from the University of Tokyo published an article in *Nature* [21] where they discovered that SCs heterogeneously express the immune checkpoint programmed death-ligand 1 (PD-L1). They found that PD-L1^+^ SCs accumulate in the body with age. Through in vitro T-cell killing assay, the researchers showed that PD-L1^+^ SCs are resistant to T cell immune surveillance, suggesting that the expression of PD-L1 in SCs is essential for evading T cell immunity. Based on these findings, the researchers tested the anti-aging effects of PD-1 antibodies. Treating naturally aged mice or mice with nonalcoholic steatohepatitis (NASH) with PD-1 antibodies significantly reduced the population of PD-L1^+^ cells in the body and improved various age-related phenotypes in a manner dependent on activated CD8^+^ T cells.

Therefore, treating the accumulation of aging PD-L1^+^ cells through ICB therapy represents a more promising treatment strategy compared to traditional anti-aging therapies.

**Figure 2:**
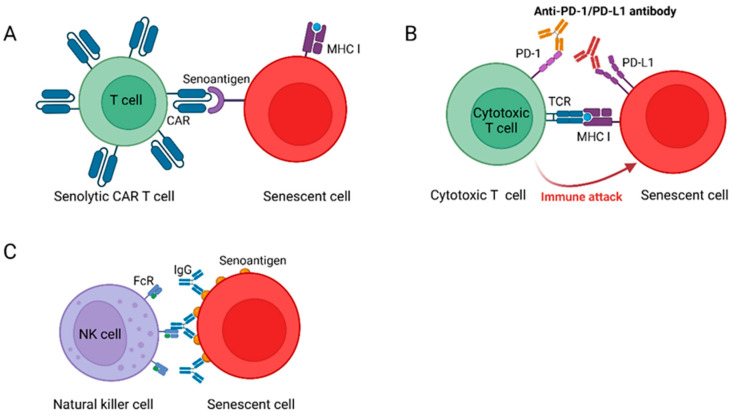
Immunotherapy targeting SCs. (**A**) Senolytic CAR T cells. Senolytic CAR T cells express chimeric receptors targeting specific antigens in SCs. (**B**) Immune checkpoint blockade (ICB). Anti-PD-1/PD-L1 antibodies attenuate the inhibitory signaling of PD-L1^+^ SCs to T cells, reactivate immune surveillance, and activate T cells to clear SCs. (**C**) Senolytic vaccination. After the injection of the senolytic vaccine, the antibodies produced by this vaccine bind only to senoantigen on the surface of SCs, thereby labeling the SCs and inducing antibody-dependent cell-mediated cytotoxicity (ADCC) to kill these SCs. Created with BioRender.com (accessed on 10 May 2024).

In summary, immunotherapy for cancer has already achieved significant initial success, with targeted therapies eliminating tumor cells and curing patients with otherwise untreatable hematological cancers [89]. Compared to the use of immunotherapy for cancer treatment, the use of immunotherapy to alleviate aging and age-related diseases may have greater advantages. By exploring the molecular mechanisms of cellular senescence and immunosenescence, researchers may identify more senoantigens, thereby developing new types of senolytics with high specificity, low toxicity, and high activity, such as senolytic CAR T cells, senolytic vaccines, and ICB (Figure 2). These immunotherapies, which clear accumulated SCs, may represent a more promising treatment strategy than traditional anti-aging therapies.

### 3.3. Other Intervention Strategies

In addition to clearing SCs, there are a range of other strategies for intervening in aging, including dietary interventions, moderate exercise, stem cell therapy, anti-inflammatory strategies, and epigenetic regulatory drugs [14,104]. These strategies have become important approaches to reverse human aging and have expanded the options for anti-aging therapies in clinical applications.

Dietary interventions, as highly actionable anti-aging strategies, play an important role by regulating metabolism, inflammation, and cellular homeostasis [105]. Caloric restriction (CR) is a widely recognized dietary intervention method in aging research that extends lifespan and delays aging. It involves reducing food intake by about 30% without causing nutritional deficiencies [106]. The benefits of CR have been observed in yeast, nematodes, fruit flies, mice, and primates [107]. Previous studies have shown that CR can improve aging-related complications such as obesity, insulin resistance, muscle degeneration, dyslipidemia, and cancer, without affecting the quality of life of the subjects [108]. Recently, research teams have discovered that CR induces various metabolic changes, including the production and circulation of metabolites [109]. Lithocholic acid (LCA) is one such metabolite that alone can replicate the effects of CR in mice, including activating AMP-activated protein kinase (AMPK), enhancing muscle regeneration, and restoring grip strength and running capacity [109]. Another study by the team revealed that LCA accumulates during CR in mammals and replicates the benefits of CR by activating the TULP3–sirtuin–v-ATPase–AMPK pathway [110]. In addition, intermittent fasting (IF) has gained attention as a new dietary intervention method in recent years. Clinical applications have found that IF can improve metabolic function and reduce body fat content [111]. Recent studies have revealed that spermidine and polyamine metabolism affect the post-translational modification of key proteins, acting as crucial control hubs for fasting-mediated autophagy and longevity [112]. Besides CR and IF, the Mediterranean diet and the Okinawan diet are also considered dietary patterns that help delay aging. The Mediterranean diet is renowned for its high proportion of plant-based foods, healthy fats (such as olive oil), fish, nuts, and moderate amounts of red wine [113]. Studies have shown that this dietary pattern is closely associated with a reduced risk of chronic diseases such as cardiovascular disease, diabetes, and Alzheimer’s disease, and can delay the aging process [114]. The Mediterranean diet is rich in antioxidants, omega-3 fatty acids, and fiber, which can alleviate oxidative stress and inflammatory responses, thus combating various pathological processes associated with aging [113]. Similarly, the Okinawan diet is widely recognized for its low-calorie, high-nutrient-density characteristics, especially in relation to the longevity and healthy aging of Okinawa’s residents [115]. The Okinawan diet emphasizes a high intake of vegetables, legumes, root vegetables, and moderate amounts of fish, while maintaining low consumption of meat and dairy products [116]. This dietary pattern not only helps control weight but also improves metabolic function, reduces inflammation levels, and is closely associated with extended lifespan and reduced incidence of aging-related diseases [116]. The longevity of Okinawa’s residents is partly attributed to the low-calorie, antioxidant-rich, and plant-based nutrient intake in their diet. Therefore, whether it is the Mediterranean diet or the Okinawan diet, both have shown significant effects in anti-aging, extending lifespan, and improving health, indicating that dietary interventions are actionable and effective anti-aging strategies by regulating metabolism, reducing inflammation, and protecting cellular functions.

In addition to Senolytics, other drugs have been found to delay aging and age-related diseases. Metformin has long been a first-line treatment for type 2 diabetes. As an oral hypoglycemic agent, it helps patients control blood glucose levels by effectively inhibiting hepatic glucose output and enhancing insulin sensitivity [117]. The primary mechanism of action of Metformin is through the AMPK pathway, regulating cellular energy metabolism and thereby improving insulin sensitivity in diabetic patients [118]. Teng Ma et al. identified the molecular target of Metformin, discovering that Metformin binds to PEN2 and initiates a signaling pathway through ATP6AP1, activating AMPK via the lysosomal glucose-sensing pathway [119]. With the extensive clinical use of Metformin, it has been found to also have anti-tumor effects [120], delay aging [121], and alleviate symptoms of neurodegenerative diseases [122]. Metformin promotes autophagic flux by regulating the level of autophagosome–lysosome fusion, significantly alleviating functional and structural changes associated with aging arteries and helping to reduce the senescence and SASP of vascular smooth muscle cells [123]. Xiaoyan Xu et al.’s research also identified Metformin as a novel activator of chaperone-mediated autophagy (CMA) and an effective drug for treating Alzheimer’s disease (AD), providing strong evidence for its role in treating CMA-related diseases [122]. In recent years, Metformin has shown potential to extend lifespan in various models, including nematodes, fruit flies, and rodents [119]. Recently, Guang-Hui Liu et al.’s research confirmed that Metformin has comprehensive anti-aging protective effects on 79 types of tissues and organs across 11 systems and can significantly enhance cognitive abilities in elderly primates, reducing biological age [121]. In summary, Metformin, as a classic hypoglycemic drug, has been proven in its extensive application in diabetes treatment. With further exploration of its mechanisms, research on Metformin in delaying aging and improving age-related conditions is gaining increasing attention, and it may become a new candidate drug for anti-aging therapy in the future.

Taurine is a semi-essential sulfur-containing amino acid that humans can synthesize, but its production is insufficient to support development during early life; hence, it is obtained from external sources [124,125]. Studies have shown that taurine supplementation is beneficial for treating metabolic and inflammatory diseases [126,127]. In June 2023, a team led by Vijay K. Yadav published an article in *Science* [128] in which they showed that the concentration of taurine in the blood of mice, monkeys, and humans decreases with age, and taurine supplementation could extend the lifespan of mice. Research on the specific underlying mechanisms revealed that taurine can protect telomerase, inhibit mitochondrial dysfunction, reduce DNA damage, and alleviate inflammatory responses. Following this, an article published by Tianyu Cao and colleagues in *Cell* also revealed that cancer cells compete with CD8^+^ T cells for taurine by overexpressing the taurine transporter SLC6A6, which induces T cell death and exhaustion. Taurine supplementation can reactivate exhausted CD8^+^ T cells and enhance the efficacy of cancer treatment [129]. These studies suggest that taurine supplementation could be an effective therapy for improving human aging and immune aging.

Additionally, supplementation with vitamin C has also been found to delay aging. Researchers discovered a novel group of CHIT1-positive microglia specifically present in the spinal cords of aged primates [130]. These cells can activate SMAD signaling in motor neurons via paracrine secretion of the CHIT1 protein, thereby driving motor neuron aging. Supplementation with vitamin C was found to inhibit the aging and degeneration of spinal motor neurons.

Recently, the concept of delaying aging through blood transfusion has attracted widespread attention. Studies on heterochronic parabiosis and rejuvenation have proven that certain components in the blood of young animals can reverse age-related decline [131]. In August 2023, Saul A. Villeda and his team identified the platelet-derived chemokine, platelet factor 4 (PF4), in plasma as a key to delaying aging in the brain. PF4 could serve as a potential therapeutic target for reducing inflammation and rescuing cognitive functions in elderly individuals [132].

Stem cells are the origin of life cells and possess characteristics such as self-replication, directed development, and the ability to repair and replace damaged cells. As stem cells continually diminish during the aging process, timely supplementation of stem cells has become a viable approach for combating aging [14]. Currently, both foundational and clinical research utilizing stem cells to alleviate aging and age-related diseases has achieved encouraging progress [133,134,135,136], and we will continue to monitor these developments.

## 4. Summary and Prospects

The global issue of population aging is becoming increasingly severe, with elderly individuals being more susceptible to infections and age-related diseases, leading to higher morbidity and mortality rates [5]. Cellular senescence and immunosenescence are closely linked to aging; therefore, this review focuses on immunotherapies targeting aging. It revisits significant recent discoveries in the mechanisms of cellular senescence and immunosenescence that have propelled the development of new treatment paradigms for aging and age-related diseases.

Traditional senolytics primarily include various natural and synthetic molecules. These small-molecule senolytics mainly inhibit SCAPs, which can lead to off-target effects that may cause damage or death to healthy cells, resulting in side effects and immune responses [137]. Moreover, the mechanisms of action for most natural senolytics have not been well defined, and their molecular targets have not been identified or characterized, making it challenging to rationally modify these compounds to enhance their senolytic activity [138]. The development of specific, low-toxicity, and high-activity senolytics is currently a challenge. Many recent studies have shown that using immunotherapies to target SCs can alleviate aging and age-related diseases, and immunotherapy-developed senolytics may be safer and more effective than traditional senolytics [139]. However, there are significant challenges in developing new senolytics through immunological means, and these challenges greatly limit the efficacy of anti-aging immunotherapeutic strategies and their further clinical application. First, our understanding of cellular senescence, immunosenescence, and the role of the immune system in the accumulation of SCs remains limited, necessitating further exploration. On the one hand, uncovering new mechanisms of cellular aging could alleviate aging by intervening in related pathways. Understanding the mechanisms of immunosenescence could help restore immune system function to combat organismal aging. On the other hand, given the notorious heterogeneity of SCs [140], universal senoantigens are unlikely to exist, and the number of identified anti-aging targets is very limited. Continued exploration of these mechanisms could help us to identify more anti-aging targets, develop new senolytics, and expand the options for senolytics in clinical applications. Second, constructing animal models is an effective method for aging research. This review summarizes the characteristics of several commonly used animal and cellular aging models (Table 1). Although significant progress has been achieved in clinical and basic aging research, many results are based on mouse models. Fundamental differences exist between the development and phenotypes of aging cells in mice and humans [141,142], and current immunological techniques and experimental progress must adequately reveal the complexity of the human immune system. Considering interspecies differences, constructing nonhuman primate and human aging models will be very important in the future. Third, considering the challenges of utilizing immunotherapy for cancer treatment in clinical settings, using immunological methods to clear SCs in clinical trials also faces a series of challenges. High levels of CAR T cell proliferation and killing of targeted cells in a short time can lead to cytokine release syndrome (CRS) [143], so it is necessary to determine the right dosage and frequency of administration that SCs can be cleared without affecting normal functions. The design of a CAR/vaccine/immune checkpoint inhibitor, the heterogeneity of SCs, the microenvironment of SCs, and the impact of combination therapies with multiple anti-aging drugs on treatment outcomes must also be considered. Moreover, SCs play important physiological roles in development, wound healing, and regeneration [36]. The memory immune cells produced by senolytic vaccines and senolytic CAR T cells can have long-lasting effects that are detrimental to normal physiological processes. CAR T cells and vaccine-induced long-term immune memory may produce off-target effects. Preclinical studies have shown that uPAR-targeted CAR T cells may attack normal cells that express low levels of this marker [19]. Therefore, it is essential to establish a long-term follow-up system to monitor the potential delayed toxicities of senolytic vaccines and senolytic CAR T cell immunotherapy, such as secondary tumors and immune exhaustion. Transient CAR T cells might be used to address these issues [139], as this would allow greater control over these undesired effects. Another issue that must be considered is the accessibility of anti-aging interventions. The high-cost, complex manufacturing processes and the cytotoxicity associated with CAR T cell immunotherapy pose challenges for its clinical application [89]. Anti-aging interventions may prioritize high-income groups, thereby exacerbating global health inequalities. Furthermore, if anti-aging technologies significantly extend healthy lifespan, they could intensify the pressures of population aging, necessitating a redesign of social security systems.

Overall, the observed effectiveness of immunotherapy-developed senolytics, particularly senolytic CAR T cells, across various aging and disease models, demonstrates their therapeutic potential, which could overcome the limitations of current senolytic drugs and potentially open new treatment avenues for various age-related diseases. The number of clinical trials involving senolytics developed through immunological means is expected to increase significantly in the coming years, but much work remains to be performed to translate these prospects into clinical treatments. We look forward to more extensive and in-depth basic research into the molecular mechanisms of cellular aging and immunosenescence, the discovery of more suitable anti-aging targets and aging models, and the promotion and expansion of senolytics in clinical applications.

## Figures and Tables

**Table 1:** Multiple models to study aging.

Model Name	Methods	Description
Cell Models		
Replicative senescence model	Repeat passages until they reach replicative senescence [22]. Human fibroblasts are replicatively senescent in their late passage (LP, *p* > 23) [23].	Cell cycle arrest, shortened telomeres, p16-positive, p21-positive, SA-β-Gal positive, and ROS increase. Negative for proliferation markers (BrdU and Ki67) [22].
Chemotherapy-induced senescence model	Postnatal NSCs were treated with 8 mM hydroxyurea for 12 h [24]. HCA2 cell senescence induction with RO3306 and nutlin3a [21].	PD-L1 positive, [21] SA-β-Gal positive, increased ROS, and increased expression of P16, p21, and p53.
Stress-induced senescence model	Fibroblasts were irradiated with UVA (5 or 10 J) and incubated for one or five days [25]. UVB treatment of melanocytes was performed at a dose of 0.125 J/cm^2^ [26]. Treatment of HDF with low doses of H_2_O_2_ leads to irreversible cellular senescence [27].	P16-positive, p21-positive, SenTraGor-positive, and SA-β-Gal-positive. Up-regulates the expression of ULBP2 and HLA-II [25].
Oncogene-induced senescence model	Human lung fibroblasts were infected with a lentivirus produced from *pTomo-KrasG12D-EGFP*/*pTomo-Teton-P16INK4a-T2A-EGFP* [28].	SA-β-Gal-positive; upregulation of p16 and NKG2DLs expression.
Animal Models		
Natural aging mice	Wild-type C57BL/6J mice cultured to 26 months [29].	Inflammatory cell accumulation, elevated levels of inflammation in the kidneys and liver, increased microglial cell infiltration, and decreased memory and learning ability.
Oncogene-induced hepatocyte senescence in mice in vivo [19,30]	Stable delivery of transposable factors expressing oncogenic *NrasG12V* into mice hepatocytes [30].	Up-regulation of p21, p16, and uPAR expression and SA-β-Gal-positive [19].
Liver fibrosis mice [19,21,31]	C57BL/6N mice were treated CCl_4_ to induce liver fibrosis [19]. Feeding mice NASH-inducing diets [31].	Liver fibrosis, liver inflammation, and upregulation of uPAR expression.
HFD-induced obese mice [20,32]	C57BL/6J mice were fed a HFD (D12492, 60 kcal% fat; Research Diets inc.) [32].	Senescent T cell accumulation, insulin resistance, increased inflammatory response, and metabolic syndrome.
Premature immunosenescence mice [33]	*Ercc1*, which encodes a crucial DNA repair protein, was selectively deleted from mouse haematopoietic cells [33].	Immune cells are susceptible to endogenous DNA damage, premature onset of immune cell senescence, significant reduction in the proportion of T cells, and impaired immune function.
Transgenic delayed aging mice [34]	Overexpression of the naked mole rat hyaluronic acid synthase 2 gene (*nmrHas2*) in mice [34].	Increased levels of hyaluronic acid, reduced inflammation and oxidative stress, decreased cancer incidence, and increased life expectancy.

LP: late passage; NSCs: neural stem cells; HCA2: human fibroblast cell line; HDF: human diploid fibroblasts; ULBP2: UL16 binding protein 2; NKG2DLs: natural killer group 2 member D ligands; uPAR: urokinase-type plasminogen activator receptor; NASH: nonalcoholic steatohepatitis; HFD: high-fat diet.

**Table 2:** Clinical trial information related to senolytics.

NCT Number	Conditions	Interventions	Clinical Progress	Starting Time	Locations
NCT04210986	Osteoarthritis	Fisetin	Phase 1/2	2020	United States
NCT05025956	Femoroacetabular Impingement	Fisetin	Phase 1/2	2021	United States
NCT04685590	Alzheimer Disease, Mild Cognitive Impairment	Dasatinib + Quercetin	Phase 2	2021	United States and Spain
NCT04063124	Alzheimer Disease	Dasatinib + Quercetin	Phase 1/2	2020	United States
NCT04815902	Osteoarthritis	Fisetin, Losartan	Phase 1/2	2021	United States
NCT05276895	Osteoarthritis	Quercetin and Fisetin	Not applicable	2022	Egypt
NCT06133634	Aging, Endothelial Dysfunction, and Arterial Stiffness	Fisetin	Phase 1/2	2023	United States
NCT06240403	Heart Failure, Systolic or Diabetes Mellitus, Type-2	Digoxin	Phase 2	2024	United Kingdom
NCT02848131	Chronic Kidney Disease	Dasatinib + Quercetin	Phase 2	2016	United States

Clinical trial registration numbers are from https://clinicaltrials.gov/ (accessed on 12 May 2024).

## Data Availability

Not applicable.
